# Prebiotic Reaction Networks in Water

**DOI:** 10.3390/life10120352

**Published:** 2020-12-16

**Authors:** Quoc Phuong Tran, Zachary R. Adam, Albert C. Fahrenbach

**Affiliations:** 1 School of Chemistry, UNSW Sydney, Sydney, NSW 2052, Australia; q.tran@student.unsw.edu.au; 2 Department of Planetary Sciences, University of Arizona, Tucson, AZ 85721, USA; zadam@email.arizona.edu

**Keywords:** autocatalysis, complexity, protometabolism, RNA, systems chemistry

## Abstract

A prevailing strategy in origins of life studies is to explore how chemistry constrained by hypothetical prebiotic conditions could have led to molecules and system level processes proposed to be important for life’s beginnings. This strategy has yielded model prebiotic reaction networks that elucidate pathways by which relevant compounds can be generated, in some cases, autocatalytically. These prebiotic reaction networks provide a rich platform for further understanding and development of emergent “life-like” behaviours. In this review, recent advances in experimental and analytical procedures associated with classical prebiotic reaction networks, like formose and Miller-Urey, as well as more recent ones are highlighted. Instead of polymeric networks, i.e., those based on nucleic acids or peptides, the focus is on small molecules. The future of prebiotic chemistry lies in better understanding the genuine complexity that can result from reaction networks and the construction of a centralised database of reactions useful for predicting potential network evolution is emphasised.

## 1. Introduction

The complexity of the cell is perhaps best indicated by the chart of intermediary metabolism [1]—an interconnected network of reactions consisting of ~500 small molecules, and derivative polymers, all within a single compartment, namely, the cellular membrane. A major challenge central to studying the origins of life is that experimental models for prebiotic reaction networks simply do not yet approach the same level of complexity that allows, for example, the cell to reproduce whilst responding to and extracting energy from its environment. This challenge may be deconstructed into three overlapping problems: (*i*) developing the tools with which to analyse the salient features of complex chemical systems, (*ii*) developing theoretical constructs of the generic features that underpin complex chemical system behaviour, in sufficient detail as to understand their dynamic modes, and (*iii*) acquiring the ability to generate entirely new complex chemical networks in the laboratory. Network analysis of enzymatic, cellular and ecological systems has led the way [2,3,4,5], advancing the development of analytical tools, which has in turn informed theory [6,7,8,9]. Nevertheless, more research into the properties of abiotic reaction networks, their generative mechanisms, and methods of analysis is still needed in order to understand how to yield the robust order associated with genuine complexity.

Applying the gains made in chemical reaction network analysis and theory to laboratory prebiotic chemistry research efforts, however, has proven difficult. A prevailing strategy [10] in prebiotic chemistry research has been to explore and refine possible chemistry within the constraints set by particular hypothetical early Earth geochemical scenarios. Typically, the aim is to ultimately produce [11] genetic or catalytic (bio)polymers thought to be important for life’s emergence. Many different synthetic pathways that connect geochemical substrates to biological compounds (or their intermediates), for example, amino- [12,13,14,15], hydroxy- [16,17,18] and keto acids [19,20,21,22,23,24], hydroxyaldehydes and sugars [25,26,27,28], nucleobases [29] as well as canonical [30,31] and noncanonical [32] nucleosides/tides have been demonstrated. While progress has been impressive, the integration of these syntheses into an interactive and compartmentalised network of molecules remains elusive. Understanding how a dynamic network of all needed synthetic pathways can come together within an enclosed volume [33,34,35,36,37] the size of the cell remains one of the primary goals of prebiotic chemistry. If this goal is to be achieved, the theory and tools of chemical reaction networks must be applied to constructively guide laboratory experimental practice. The topological attributes of biotic reaction networks elucidate the structural underpinnings of what is observed as emergent behaviour in biology—resolving these attributes in the context of abiotic reaction networks ought to be advantageous for characterising the early chemistry that led to biology [9].

From a purely chemical perspective, the study of reaction networks also offers a comprehensive map [38] of the different pathways that can yield relevant molecules from available feedstocks. In complex networks, there may be multiple possible pathways, each with different aggregate rates and timescales, to synthesise a compound from an array of substrates. Modelling reaction networks enables a means of quantifying the prebiotic plausibility of producing a target compound, particularly in cases where the most efficient pathway may not dominate over the long-term, if there are competing pathways that utilise a limiting intermediate. While biological reaction networks that govern extant metabolism may bear no resemblance to their progenitors, nevertheless, the study of abiotic reaction networks generally overlaps with the research aims sought after by the field of systems chemistry [39,40], namely, emergent chemical behaviours, autocatalysis, self-organisation and replication—all of which are accompanied by out-of-equilibrium mechanisms and dynamic fluctuations.

This review provides a focused update on some of the more recent experimental reports detailing model prebiotic reaction networks that occur in liquid water. The aim is to give a brief discussion of (*i*) the importance of reaction networks, (*ii*) to provide some historical context of their use, and then (*iii*) to highlight some contemporaneous studies of potentially prebiotic reaction networks that either give rise to important building blocks, employ atypical analytical techniques or otherwise demonstrate novel systems-level behaviour. This review will not dwell on gas- or solid-phase chemistry, or networks utilising alternative solvents such as formamide, although networks employing dry-down of the aqueous phase which may lead to reactions in a remnant organic liquid will be covered. Discussion of reaction networks involving nonenzymatic oligo- and polymerisation of nucleic acids, peptides or similar structures also will be avoided, focusing instead on small molecules.

## 2. What Is a Chemical Reaction Network?

According to the *Encyclopedia of Astrobiology* [41], “A chemical reaction network consists of a set of chemical reactions and a set of chemical compounds. Each chemical compound is a node of the network. Each chemical reaction is a directed vertex of the network, connecting the chemical compounds involved in the reaction, from the reactants towards the products.” This definition encompasses multiple extrema of chemical transformations—e.g., from simple acid-base and bimolecular reactions between a handful of compounds on one end, to extremely complex maps of thousands of macromolecule-catalysed and -mediated interactomes of metabolism or genetic transcription and translation on the other. Nevertheless, the generalised notion of how one may construct testable hypotheses to evaluate a reaction network in the context of prebiotic chemistry enjoys a more intuitive connotation, at least in terms of defining its characteristic complexity. In other words, not unlike the categorisations used in relation to obscenity given by Justice Potter Stewart of the United States Supreme Court, prebiotic reaction networks have an “I know it when I see it” [42,43] quality about them. They are not meant to refer to simple acid-base transformations, or even acid-base catalysed covalent reactions *per se*. Rather, a certain level of complexity is implicated and expected, but what distinguishes genuinely “complex” from merely “complicated” chemistry is still a matter of active discussion.

A number of straight-forward features have been predicted [9] to be characteristic of plausible prebiotic chemical reaction networks. One of the most fundamental properties of a network is its degree distribution, wherein degree is defined as the number of connections (reactions) to other nodes (molecular members of the chemical network). For prebiotic networks, at least one node should serve as a hub, i.e., a node of above-average degree. At a minimum, a hub should exist that possesses at least two outgoing connections to other nodes via covalent bond transformations that are not just simple Brønsted acid-base reactions. That is to say, some molecules of the network will be common substrates or intermediates for many reactions, a feature which potentially allows a variety of products to ensue from relatively simple starting conditions [9]. Complex chemical networks also afford [9] a form of robust buffering capacity—if any one chemical member is lost or depleted, the chances of systemic persistence are enhanced by the interconnectedness of the network surrounding that member. Closed cycles (series of reactions that form a loop of inputs and outputs) are another feature typically predicted for prebiotic reaction networks, wherein the entire cycle potentially can be generated starting from any chemical member along that cycle given appropriate conditions. Closed loops within a network create feedback mechanisms that enable internal regulation and persistence amidst fluctuating environmental circumstances. A “viable core” results when a closed loop consists of molecules that can mutually catalyse their own formation in a self-sustaining fashion [9]. It is important to note that biological reaction networks have also been characterised [44,45] by similar sorts of network-level attributes. For example, rather than being randomly connected networks, wherein most nodes on average have the same degree, metabolic networks exhibit heterogeneous connectivity distributions (e.g., power law, lognormal, exponentially truncated power law, etc.). That is to say, there exists a few nodes with a very large number of connections, and a large number of nodes with only few connections. Other important network-level features [46] of biochemical networks include the shortest average pathlength, average clustering coefficient and assortativity. A recent study by Walker and co-workers [45] analysed biochemical networks for these features at the level of individuals, ecosystems and the entire biosphere. The authors found that some of these features are distributed according to the same scaling laws across all three levels. Whether such scaling laws also characterised the prebiotic reaction networks responsible for life’s origins remains unknown; meanwhile, serious criticisms [47,48] regarding the statistical significance and mechanistic interpretation of purported scaling laws in a variety of contexts not limited to biochemistry have also been raised.

From an experimental perspective, the degree of human intervention [49] required to connect disparate reactions with one another into a realised chemical system further categorises model prebiotic reaction networks, and helps to identify potentially applicable geochemical scenarios. This attribute exists along a spectrum, with “continuous” on one end and “discontinuous” on the other. Discontinuous models for prebiotic synthesis have been described previously [50] as requiring discrete intervention steps, employing for example specific orders of additions of reagents, or specific steps in which some compounds are isolated, concentrated, purified or removed to create conditions that permit subsequent reactions to occur. Continuous reaction networks, on the other hand, have been defined [51] as those which minimise or entirely remove human interference, wherein for example, timely additions of reagents are not carried out, neither are purification or removal steps. It is important to note that “discontinuous” does not automatically mean “geochemically implausible”, since some discontinuous synthesis models may be accurate representations of specific scenarios with discrete phase changes or abrupt transitions. In addition, just because a reaction network was constructed or carried out in a discontinuous fashion does not automatically imply such a network has no chance of occurring in a more continuous manner. Nevertheless, it is advocated herein that the continuous/discontinuous classification, strictly speaking, is one that refers to the nature of the experimental laboratory protocols that are employed to achieve the reaction network under study. That is to say, reaction networks themselves are not intrinsically continuous or discontinuous—these descriptors only describe how such reaction networks were achieved in the lab or otherwise constructed on paper or *in silico*. Moreover, where a reaction network lies along the continuous-discontinuous spectrum has also been implicated in discussions of “metabolism first” versus “genetics first” approaches [52] to the origins-of-life problem. In particular, it has been argued [53] that genetics-first hypotheses, like the RNA world, are more tolerant to discontinuous scenarios, whereas metabolism-first arguments tend to put greater emphasis on the importance of continuous reaction networks. While not a comprehensive review, notable recent prebiotic reaction networks ranging among these different salient features and categories will be highlighted.

## 3. The Miller-Urey Experiment

**Figure 1:**
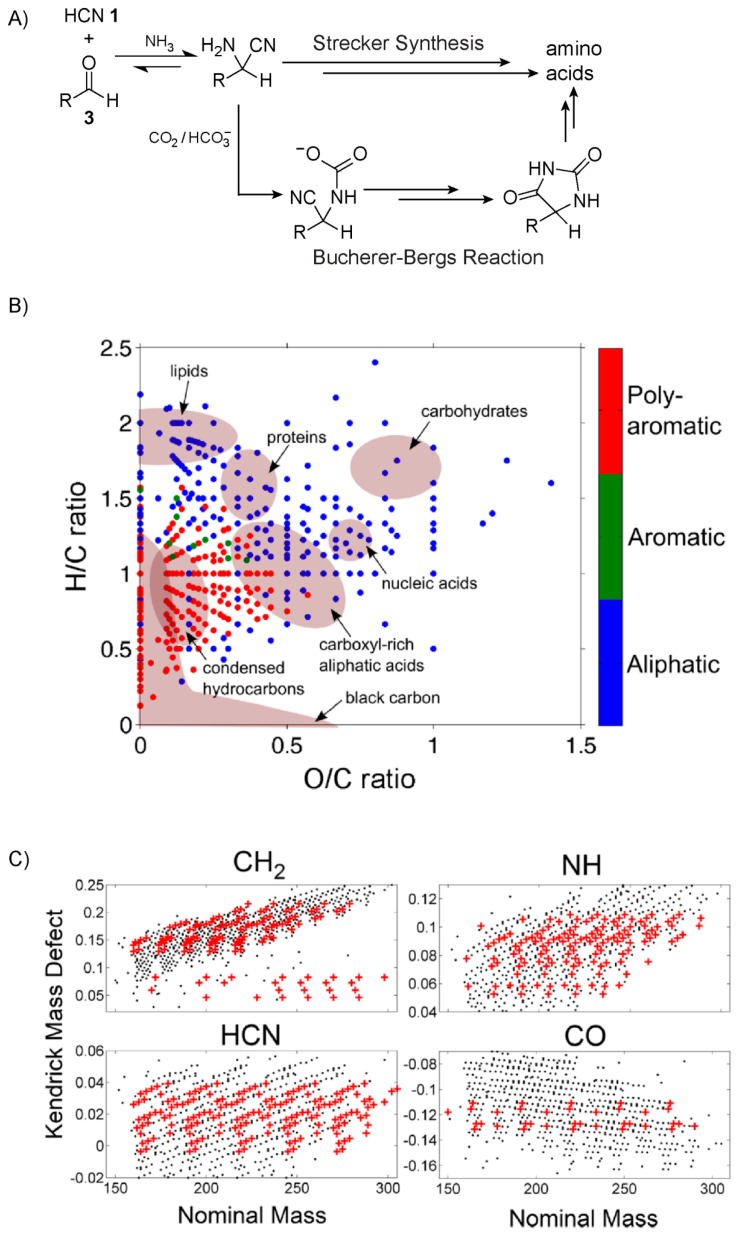
Proposed mechanisms and data analysis plots for the Miller-Urey experiment. (**A**) Two pathways proposed for amino acid production, namely Strecker and Bucherer-Bergs syntheses, which rely on the formation of α-aminonitriles. While the Strecker synthesis relies on slow hydrolysis of aminonitriles, Bucherer-Bergs relies on the more efficient hydrolysis of hydantoins formed from reaction between CO_2_ and aminonitriles. (**B**) Van Krevelen diagram of Miller-Urey products categorised by their H/C versus O/C ratios. (**C**) Kendrick maps displaying homologous series of CH_2_, HCN, NH, and CO. Plots in (**B**,**C**) reprinted with permission from reference [66].

The Miller-Urey experiment has been credited [54] as the first intentional demonstration of prebiotic synthesis, which historically helped to initiate the modern era of prebiotic chemistry research. This experiment is an example of a complex reaction network involving both gas- and aqueous-phase reactions. In 1953 [55], in order to test the hypothesis put forth by Oparin [56] and emphasised by Urey [57] and Bernal [58] that the organic compounds important for life were formed from a reducing atmosphere, Stanley Miller carried out his eponymous electric spark-discharge experiment which simulated atmospheric lightning. These reducing gases were considered the major components of the early Earth atmosphere. The current consensus, however, is that the early atmosphere as a stable steady state most likely would have been largely neutral, comprised primarily of CO_2_ and N_2_, although more reducing atmospheres could have existed transiently [59,60]. In order to simulate lightning in a primitive atmosphere, electrodes delivered spark-discharges to a gaseous mixture of CH_4_, NH_3_, H_2_O, and H_2_. Hydrogen cyanide (HCN) **1** and aldehydes were some of the more prevalent products formed from this gas-phase chemistry [61,62] (numbers given in bold refer to specific compounds which are displayed in the Figures). In the proposed mechanism, these molecules then rain into an aqueous reservoir where they continue to react, generating a wide variety of products including, famously, amino acids. Mechanisms for the production of amino acids have been proposed [63], for example the Strecker reaction [64] and the Bucherer-Bergs pathway [65] which exploits hydantoin intermediates. For more details, see Figure 1A and the caption.

Amino acids, however, represented only a small fraction of the total number of compounds that were being produced from this reaction network. The Miller-Urey experiment has been revisited many times over the years, and the full extent of products formed from this classic reaction network are still being investigated. In 2016, Wollrab, Ott and co-workers [66] set out to further characterise the chemical variety of these Miller-type prebiotic “broths”. Rather than taking the traditional specific-target-focused approach of identifying potentially important prebiotic compounds like amino acids, these researchers aimed to carry out a broader overview of all the species being synthesised. Two basic variants of the Miller-Urey experiments were performed, one in which spark-discharge took place in the gas-phase and another in which spark-discharge occurred between the electrode and the surface of the aqueous phase. The authors noted that for each case, the results were similar. The chemical composition of the aqueous phases was then analysed using electro-spray ionisation high-resolution mass spectrometry and NMR spectroscopy. Close to 700 unique molecular formulas, e.g., C_5_H_7_N_3_O_2_, C_8_H_10_N_2_O_2_, and C_9_H_6_N_6_, were measured by mass spectrometry over the range of 150 to 300 *m*/*z*. In order to analyse this large number of molecular formulas, chemical indices and graphical analysis tools, like Van Krevelen diagrams [67] and Kendrick maps [68], were applied to the data sets.

Categorisation of double bond equivalents as well as aromaticity indices revealed a broad distribution in the degree of saturation and aromaticity of the compounds observed. Van Krevelen diagrams, which plot a compound’s hydrogen/carbon ratio against its oxygen/carbon or nitrogen/carbon ratio, can further help classify the types of molecules being formed. For example, carbohydrates, proteins, lipids and nucleic acids all occupy distinct regions of these diagrams. This analysis further confirmed that the Miller-Urey broth contained a wide-spectrum of compounds (Figure 1B). Kendrick maps allow for the easy identification of homologous series of molecules, and the authors found that the largest groups of these molecules were derivatives of CH_2_, NH, HCN and CO (Figure 1C). Homologous series of CH_2_ are indicative of alkanes, the presence of which was further confirmed by NMR spectroscopy. The oligo- and polymerisation of HCN is well known [69], chemistry of which is capable of forming, for example, the adenine nucleobase [70]. The CO homologous series suggests the presence of ketones, aldehydes and carboxylic acids. Unexpectedly, evidence for the formation of short polyethylene glycol oligomers was also observed.

In a follow-up study by Scherer, Ott and co-workers [71], the authors noted that even though the starting conditions of some of the Miller-Urey experiments they conducted were as similar as possible across separate runs, there was high variability in terms of the distribution of end products observed by NMR spectroscopy. The authors suspect that the reaction network is of such complexity with thousands of interconnected members, some of which may be serving as catalysts, the outcome in terms of the final product distribution is nonlinear. That is to say, different distributions of products may result even though the initial conditions are virtually identical, a feature reminiscent of biological systems [72].

## 4. Hydrogen Cyanide Polymerisation

While the fact that HCN **1** can polymerise under aqueous conditions is well known [69], the details of the ensuing reaction network are not fully understood. HCN polymerisation leads to dark insoluble precipitates. The structure of these polymeric precipitates has not been completely elucidated, but several proposals [73,74,75] have been made. Whatever the exact structure, hydrolysis of the resulting polymers can afford molecules of prebiotic interest, including amino acids, purines and pyrimidines [69]. Thus, the complexity of HCN polymer chemistry can serve as a useful model for studying chemical reaction network evolution.

The vast number of compounds produced, like in the case of the Miller-Urey-type experiments, however, poses major challenges during analysis. Experimental combined with computational methods can be a powerful way to gain insight into complex chemistry. One computational approach [76,77,78] towards understanding complex reaction networks, which assists in the prediction of possible molecules synthesised and their potential chemical pathways, works by representing compounds abstractly as mathematical objects known as graphs. Graph grammar rules, which encode specified reaction mechanisms and instruct the network how to evolve can then be applied, affording computational enumeration of all combinatorial possibilities within a selected limit.

**Figure 2:**
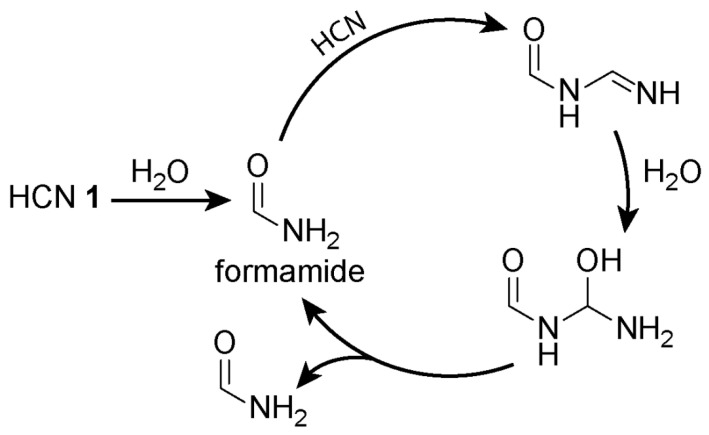
A proposed autocatalytic loop that revolves around the formamide-catalysed hydration of HCN. Formamide, first produced from HCN hydration, can subsequently serve as a catalyst for its own production [79].

In 2013, Merkle and co-workers [79] demonstrated a strategy for investigating HCN **1** polymerisation as a model complex reaction network by employing mass spectrometry coupled with theoretical explorations of the possible chemical space using graph grammars. The team first carried out HCN polymerisation, and then the polymeric precipitates were subjected to a variety of different hydrolytic conditions. The supernatants containing the soluble hydrolysis products then were analysed by mass spectrometry. Next, theoretical exploration of the chemical space was carried out computationally using a set of appropriate graph grammar rules that reflect known reaction mechanisms of HCN **1** polymerisation and hydrolysis. The resulting compounds generated theoretically then were filtered according to the molecular ions determined experimentally by mass spectrometry. Molecules and pathways consistent with high intensity peaks observed in the mass spectra were given a high preference. Additional filters were applied, i.e., excluding reactions that happen between relatively large molecules and eliminating unlikely compounds based on calculated free energies. After filtering, 94 out of 6,472 candidate compounds that were generated computationally from graph grammar iteration remained. To demonstrate the explorative potential of this approach, the authors highlighted possible pathways to adenine synthesis which are alternatives to the one proposed by Oró [70,80,81]. A potential autocatalytic loop (Figure 2) that revolves around the formamide-catalysed hydration of HCN **1** was also identified. This type of close coupling between experiments and computational modelling offers a powerful and promising way to gain a better understanding of complex chemical spaces.

## 5. The Formose Reaction

While the Miller-Urey experiment is famous for production of amino acids, to date, *de novo* synthesis of ribonucleosides/tides has not been reported from this type of reaction network (although it is important to note that the production of nucleosides from irradiation of formamide has been reported [82]). The same can be said about carbonaceous chondrite meteorites, like the Murchison. Analysis of its soluble organic matter extracts has revealed [83,84] a vast chemical inventory but no detectable nucleosides or derivatives thereof (both sugars [85] and nucleobases [86], however, have been detected). Prebiotic RNA synthesis has been a central topic of origins-of-life research as a consequence of its relevance to the RNA world hypothesis [87,88], and more generally, genetics-first models of abiogenesis. An RNA world prior to the arrival of the last universal common ancestor is often invoked [89] to explain the origin of the ribosome as well as the use of ribonucleotide-derived enzyme co-factors [90]. Taken to its extreme, the RNA world hypothesis suggests that RNA or something closely related was the original genetic polymer that emerged from early Earth geochemistry, setting the stage for the beginnings of Darwinian evolution through which the rest of metabolism was realised. Decades of research, however, have demonstrated that prebiotic synthesis of ribonucleotides, generally speaking, is substantially more difficult in comparison to amino acids. Ribonucleotides have been dubbed the “prebiotic chemist’s nightmare” [50,91] as a consequence of their relatively complex stereochemistry and thermodynamically unstable bonds, but nevertheless, a number of possible discontinuous prebiotic synthetic strategies have been proposed and tested experimentally.

One general synthetic strategy involves the separate formation of ribose **2** and nucleobase components, followed by *N*-glycosidic bond formation. In this context, the formose reaction has been cited often as the prebiotic source of ribose. First discovered by Butlerow in 1861 [92], the formose reaction is a reaction network that arises from formaldehyde **3** given the production of trace amounts of glycolaldehyde **4**, and in the context of prebiotic chemistry is typically carried out at basic pH, elevated temperatures and Ca^2+^ ions. This reaction network produces a large variety of aldoses and ketoses, including ribose. These carbohydrates are built up primarily through a series of aldol additions starting from glycolaldehyde and formaldehyde. At least trace amounts of glycolaldehyde are required to initiate the reaction, which forms an enolate that serves as the nucleophile in the first aldol reaction. Breslow later showed [93] that this reaction network is autocatalytic by the fact that the tetroses formed can undergo retroaldol cleavage to yield two molecules of glycolaldehyde for each one consumed.

The formose reaction has since received wide attention from the prebiotic research community as a potential source of sugars on the early Earth. Versions of the formose reaction have even been carried out within vesicle compartments [94]. While the formose reaction is cited often in prebiotic chemistry, its limitations also have been highlighted [95], and are summarised as follows: (*i*) the mixture of products is complex with no preference for ribose (yield < 1%), and (*ii*) once formaldehyde is completely consumed, known as “the yellowing point”, the conditions that initially afforded the reaction network then lead to the decomposition of the very products generated.

**Figure 3:**
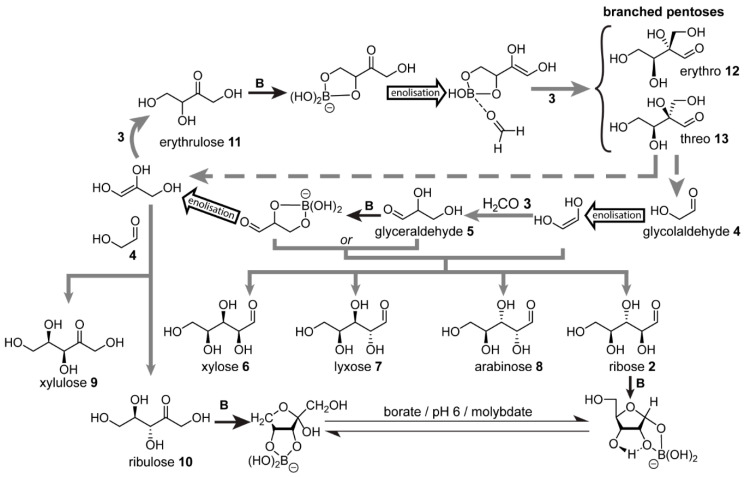
Simplified scheme of borate-mediated formose reaction proposed by Benner and co-workers [50,96,97]. The main reaction sequence produces aldoses and ketoses of increasing complexity following a series of aldol additions with formaldehyde starting from glycolaldehyde. Branched pentoses **12** and **13**, which cannot further enolise, undergo retro-aldol fragmentation to produce glycolaldehyde and glyceraldehyde or molybdate-catalysed Bilik conversion (reaction not shown) to linear ketoses **9** and **10**, respectively. In presence of molybdate, ribulose equilibrates to ribose. **B**: borate. Compounds are depicted as d- or l-isomers but exist as racemic mixtures.

In order to circumvent these limitations, Benner and co-workers proposed [50,96,97] a borate ([B(OH)_4_]^−^)-mediated formose scenario (Figure 3), which exploits borate’s ability to form complexes with 1,2-diols (also known as vicinal diols). These variations were investigated in the presence of borate buffer under otherwise typical prebiotic formose conditions, i.e., formaldehyde **3**, variable amounts of glycolaldehyde **4** and Ca^2+^ cations, basic pH and elevated temperatures. The aldol addition of glycolaldehyde with formaldehyde is the first step, yielding glyceraldehyde **5**. The team showed that when the initial glycolaldehyde and formaldehyde concentrations are comparable, the glyceraldehyde formed, which weakly complexes with borate, reacts as the electrophile with the glycolaldehyde-derived enediolate to form a mixture of aldopentoses, namely, xylose **6**, lyxose **7**, ribose **2**, and arabinose **8** [50]. Alternatively, when the respective roles of glycolaldehyde **4** and glyceraldehyde **5** as nucleophile and electrophile reverse, the products are xylulose **9** and ribulose **10** [50]. If formaldehyde **3** is in excess, however, as is typical for the classical formose reaction, glyceraldehyde will serve as the nucleophile after enolisation. Reaction with formaldehyde then takes place, forming a new C–C bond at the less-hindered terminal carbon, resulting in the ketose erythrulose **11**. This ketotetrose then undergoes borate-mediated enolisation and aldol addition with formaldehyde to form the erythro- and threo-branched pentoses **12** and **13**, respectively, which form stable borate complexes and cannot undergo further enolisation due to the lack of hydrogen at the α-carbons. At high pH, it is unlikely that these branched pentoses will decomplex with borate. If they could, however, the team showed that retroaldol cleavage would immediately take place to yield glyceraldehyde enediol and glycolaldehyde. Benner and co-workers proposed that if the pH of the mixture were to decrease, possibly due to carbonic acid derived from atmospheric CO_2_ in the form of acidic rain precipitation, the borate complexes would weaken, allowing the branched pentoses to dissociate [97]. Under slightly acidic conditions (pH ~ 6), molybdate (Mo$O_{4}^{2–}$) was shown to catalyse the Bilik reaction which rearranges the branched pentoses **12** and **13**, giving, respectively, the linear ketopentoses xylulose **9** and ribulose **10**. These ketoses subsequently equilibrate in the presence of molybdate to give the corresponding aldopentoses, namely, xylose **6** and ribose **2** [97]. Hence, this borate-mediated reaction network enables carbohydrates useful for prebiotic chemistry to undergo both synthesis and accumulation.

With the borate-mediated formose reaction network as the presupposed source of ribose **2** [96], Kim and Benner more recently investigated the dry-down synthesis [98] of purine (adenine, hypoxanthine and 2,6-diaminopurine) and pyrimidine (pyrimidin-2-one) ribonucleotides via direct ribosylation using ribose-1,2-cyclic phosphate, synthesised according to methods detailed by Krishnamurthy and co-workers [99]. The free nucleobases, meanwhile, potentially could have formed from either HCN **1** [70,100] or formamide [101]. (It is worth mentioning that mixtures of phosphoric acid, d-ribose and uracil have been shown [102] to also afford *N*-glycosidic bond formation in a proposed mechanism that proceeds through ribose-1-phosphate as an intermediate, given the mixtures are first transformed into microdroplets. For more details, see the reference cited [102]).

## 6. Reaction Networks for Ribonucleotide Synthesis

The reason Benner and co-workers rely on ribose-1,2-cyclic phosphate is that otherwise direct *N*-glycosidic bond formation between ribose **2** and either purines or pyrimidines leading to the canonical β-furanoside isomers is inefficient at best. In the case of the pyrimidines cytosine and uracil, ribosylation does not work partly because the *N*-1 lone pairs are delocalised across the ring systems, rendering them poorly available for nucleophilic attack. For the purine adenine, a mixture of isomers results from multiple nucleophilic reaction sites on the nucleobase, the presence of pyranose and furanose forms of ribose, as well as lack of stereoselectivity at the anomeric carbon. Ribose-1,2-cyclic phosphate employed by Kim and Benner helps to solve these issues through its chemically activated structure which fixes the furanose form of the ring, and at the same time, favours the production of the β-anomers.

Another strategy for prebiotic synthesis of both the pyrimidine and purine [103,104] ribonucleotides, which makes use of preformed ribose **2** presumably generated from a formose-type reaction, was demonstrated in 2019 by Carell and co-workers [105]. Instead of using a fixed, activated structure like ribose-1,2-cyclic phosphate, their approach relies on *N*-glycosidic bond formation with ribose using purine and pyrimidine nucleobase precursors that display reactive exocyclic amines; after ribosylation through dry-down, the constructions of the nucleobases are finalised, furnishing the canonical ribonucleosides regioselectively albeit among other α and pyranoside isomers.

Building on their previous work [103,104] on prebiotic purine ribonucleotide synthesis, the authors propose a model geochemical reaction network capable of yielding both pyrimidine and purine ribonucleotides beginning from a common mixture of small molecule substrates. The reaction network relies on wet-dry cycles and well-timed additions of reagents that might occur given a particular set of geochemical circumstances. The production of the pyrimidine and purine precursors, namely isoxazolylurea and formamidopyrimidines, respectively, occurs in a separate location from the geochemical source of ribose. These nucleobase precursors once formed then combine with ribose towards the end of the synthetic sequence, reacting to form the *N*-glycosidic bond, followed by completion of the nucleobase structures and phosphorylation.

**Figure 4:**
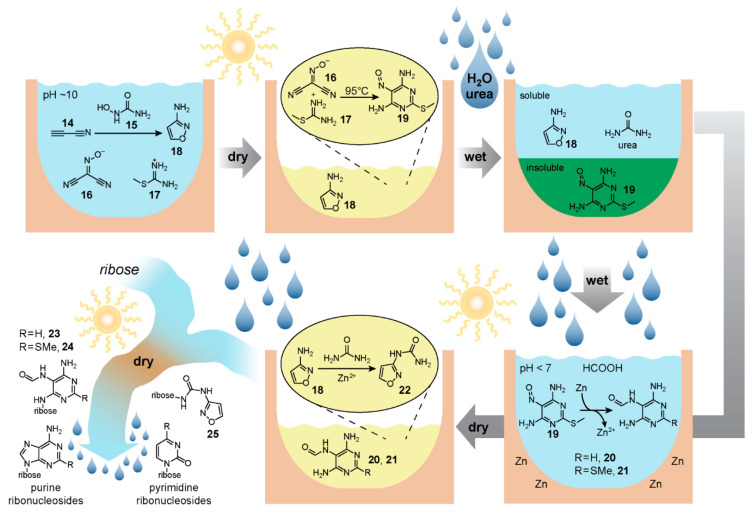
Geochemical scenario for mutual synthesis of purine and pyrimidine ribonucleotides proposed by Carell and co-workers [105]. An initial mixture of cyanoacetylene **14**, (hydroxyimino)malononitrile **16**, hydroxylurea **15**, and methylthioamidine **17** goes through a series of wet-dry cycles which relies on the separate delivery of urea and formic acid via rain as well as Zn/Zn^2+^ chemistry to produce pyrimidine and purine nucleobase precursors **20**–**22**. A stream carrying these precursors then merges with another carrying ribose, and a subsequent wet-dry cycle drives the coupling reactions. The figure image was redrawn based on Figure 5B of reference [105].

The reaction network begins [105] (Figure 4) with an aqueous mixture of cyanoacetylene **14**, hydroxylurea **15**, (hydroxyimino)malononitrile **16**, methylthioamidine **17** and sodium carbonate at pH 10. First, cyanoacetylene, which can be derived [106] from spark-discharge of gaseous mixtures of methane and nitrogen for example, cyclises with hydroxylurea to form 3-aminoisoxazole **18**, a liquid in neat form with a high boiling point. In the proposed geochemical scenario, removal of water by dry-down takes place next, allowing 3-aminoisoxazole to essentially act as an organic solvent that facilitates the reaction of the still-present (hydroxyimino)malononitrile **16** and methylthioamidine **17** affording 2-(methylthio)-5-nitrosopyrimidine-4,6-diamine **19**, an intermediate *en route* to purine ribonucleotide synthesis. Subsequently, the reaction mixture is re-wetted, with urea delivered at the same time, possibly via rain. The 2-(methylthio)-5-nitrosopyrimidine-4,6-diamine **19** is insoluble in the aqueous phase, potentially allowing for separation of the supernatant containing 3-aminoisoxazole **18** and urea into a separate nearby reservoir. The insoluble nitrosopyrimidine **19**, on the other hand, can then perhaps combine with dilute formic acid (also maybe delivered by rain), and metallic zinc from the Earth’s crust. The zinc acts as a reducing agent and together with formic acid yields formamidopyrimidines **20** and **21**—purine nucleobase precursors that can form *N*-glycosidic bonds with ribose. If this mixture then combines back with the aqueous mixture of 3-aminoisoxazole **18** and urea in the separate reservoir, subsequent dry down yields, through a reaction catalysed by the Zn^2+^ formed in the previous step, isoxazolylurea **22**—a pyrimidine nucleobase precursor also capable of reaction with ribose.

Assuming at this stage the geochemical scenario can afford the delivery of ribose along with boric acid, further wet-dry cycles can drive *N*-glycosidic bond formation affording the β-furanoside intermediates **23**, **24** and **25** along with a mixture of other α and pyranoside isomers. While this dry down-step after re-dissolving in basic water and heating also finishes the construction of the purine ribonucleosides, an additional reduction step plausibly catalysed by iron(II) in the presence of thiols is needed for the completion of the pyrimidine nucleobase from compound **25**. Another dry-down in the presence of urea and a phosphate-containing mineral such as lünburgite affords phosphorylation to the canonical purine and pyrimidine ribonucleotides. Although this proposed geochemical scenario relies on a number of well-timed additions and separations, the authors demonstrate a reaction network with unique mechanisms for ribonucleotide synthesis that takes full advantage of the various intermediates and side-products formed along the way.

**Figure 5:**
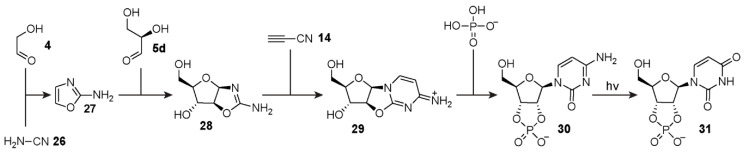
Activated pyrimidine ribonucleotide synthesis demonstrated by Sutherland and co-workers [108]. The first step involves the reaction between glycolaldehyde **4** and cyanamide **26**, which after cyclisation, yields 2-aminooxazole **27**. d-glyceraldehyde **5d** reacts with **27** to form d-arabinose aminooxazoline **28** among other stereoisomers (not shown). Compound **28** further reacts with cyanoacetylene **14** to yield 2,2′-anhydrocytidine **29**. Phosphorylation of **29** yields cytidine-2′,3′-cyclic phosphate **30**, which can undergo UV-promoted deamination to form uridine-2′,3′-cyclic phosphate **31**.

Another alternative strategy for ribonucleotide production that circumvents the inefficient *N*-glycosidic bond formation between ribose and nucleobases has been demonstrated by Sutherland and co-workers [107,108]. Building on the work of Sanchez and Orgel [109], the Sutherland group uncovered a synthetic protocol whereby both the sugar and pyrimidine nucleobase components are formed concurrently, avoiding the need for *N*-glycosidic bond formation directly from ribose **2**, as well as the separate production of ribose altogether. In 2009 [108], the team demonstrated a series of reactions commonly known as the Powner-Sutherland pathway (Figure 5) that yields the pyrimidine ribonucleotide-2′,3′-cyclic phosphates. This synthesis employs relatively simple organic reagents that plausibly were available on the early Earth, namely, cyanamide **26**, glycolaldehyde **4**, d-glyceraldehyde **5d**, and cyanoacetylene **14** as well as inorganic phosphate (P_i_). The Powner-Sutherland pathway starts from the cyclisation of cyanamide with glycolaldehyde to form 2-aminooxazole **27** in the presence of P_i_ at neutral pH. Next, another cyclisation reaction occurs after the addition of 2-aminooxazole **27** to d-glyceraldehyde **5d**, forming a mixture of pentose aminooxazoline stereoisomers. This mixture is primarily composed of the d-arabinose **28** and d-ribose aminooxazolines with smaller amounts of d-xylose and d-lyxose derivatives, the latter of which is in equilibrium with its furanose and pyranose isomers. The d-arabinose aminooxazoline **28**, which is required for canonical ribonucleotide synthesis, then reacts with cyanoacetylene to produce 2,2′-anhydrocytidine **29**. This product can then be phosphorylated to yield cytidine-2′,3′-cyclic phosphate **30**. Subsequent irradiation by UV-light promotes deamination of cytosine to afford the other canonical pyrimidine ribonucleotide, namely, uridine-2′,3′-cyclic phosphate **31**. In a recent publication, this pathway was further elaborated to include the synthesis of purine deoxyribonucleosides [110].

**Figure 6:**
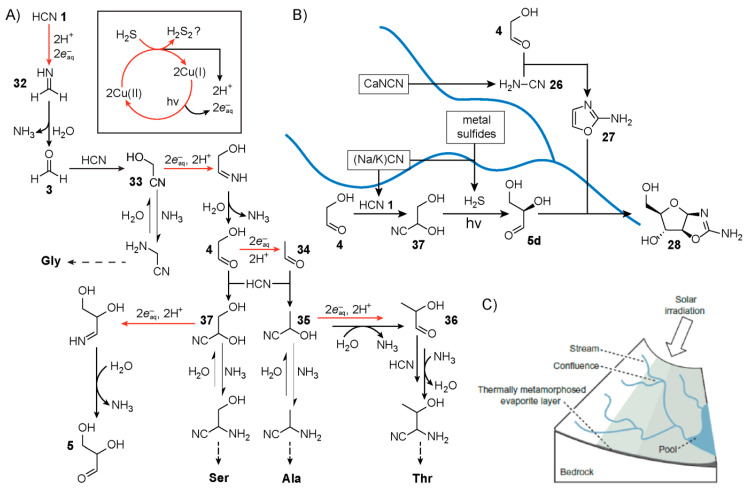
Cyanosulfidic geochemical scenario proposed by Sutherland and co-workers [26,111,112]. (**A**) Reaction scheme for the cyanocuprate-photocatalytic synthesis of ribonucleotide and amino acid intermediates. Not shown are pathways for additional amino acid and phospholipid precursors. For more details, see reference [112]. (**B**) Scheme for arabinose aminooxazoline synthesis **28** via the Powner-Sutherland pathway from potential evaporites that could have yielded the necessary starting materials. Image redrawn based on Figure 2D of reference [112]. (**C**) A post-meteoritic impact geochemical scenario where streams produced from rainfall carry different starting materials derived from various evaporites as shown in (**B**). The image in (**C**) was reprinted with permission from reference [112].

While much of the original pathway reported by Sutherland and co-workers was carried out in a stepwise (discontinuous) fashion, in 2012, Ritson and Sutherland [26] revealed a more continuous reaction network starting from HCN **1** that produces glycolaldehyde **4** and glyceraldehyde **5** in a chemical process driven by UV-photocatalytic irradiation of cyanocuprate complexes (Figure 6A). The production of these hydroxyaldehydes follows a Kiliani-Fischer homologation mechanism that occurs within a single aqueous mixture. First, irradiation of the cyanocuprates photooxidises the copper(I) centres to copper(II) generating in the process a hydrated electron ($e_{aq}^{-}$), which effectively serves as a reducing agent. The HCN **1** in solution is then reduced to methanimine **32**, likely proceeding through a methaniminyl radical intermediate. Methanimine is subsequently hydrolysed by water to form formaldehyde **3**, releasing an equivalent of ammonia. Reversible addition of HCN to formaldehyde yields the cyanohydrin glycolonitrile **33**, which like HCN **1** can also undergo reduction affording the imine that yields glycolaldehyde **4** after hydrolysis. Another round of this homologation chemistry yields a racemic mixture of glyceraldehyde **5**. Meanwhile, the copper(II) complexes oxidise HCN to cyanogen thereby restoring the initial copper(I) state. Spontaneous hydrolysis of cyanogen yields cyanate, which reacts irreversibly, however, with both glycolaldehyde and glyceraldehyde to form oxazolidinone rings. This outcome puts a cap on the open-endedness of this reaction network, which limits the usefulness of these hydroxyaldehydes for ribonucleotide synthesis, at least in this specific context.

Howbeit, Ritson and Sutherland later showed [111] that addition of hydrogen sulfide to the initial mixture could serve as the sacrificial reductant instead of HCN **1** to restore the copper(I) state. The inclusion of H_2_S thus circumvents the production of cyanate in the reaction network, leaving the hydroxyaldehydes free for further potential downstream reactions (Figure 6A). Acetaldehyde **34**, a product of glycolaldehyde **4** reduction, was also observed, as was its cyanohydrin namely, lactonitrile **35**. In the presence of ammonia, these cyanohydrins equilibrate to their α-aminonitrile counterparts, which serve as precursors for amino acid synthesis. Lactonitrile can also yield lactaldehyde **36** after nitrile reduction, which in the presence of HCN **1** and NH_3_, equilibrates to the aminonitrile precursor for (allo)threonine.

In 2015, Sutherland and co-workers [112] built upon this work by demonstrating the synthesis of additional amino acids and phospholipid precursors by employing a set of common mechanisms, including Kiliani-Fischer, H_2_S addition to nitriles, UV-photoreductions as well as copper-catalysed cross coupling. The authors proposed [112] a cyanosulfidic geochemical scenario (Figure 6B) based upon meteorite impact capable of affording, in particular, a chemical network for the Powner-Sutherland pathway. Model studies of meteorite impacts have shown [113] the gas-phase production of HCN. Meteorites could have also delivered important iron- and phosphorus-containing [114,115] metallic minerals. The HCN produced by meteorite impact could have been then sequestered by coordination with ferrous iron yielding ferrocyanide salts. Further thermal processing of these salts may have led subsequently to the availability of the necessary organic precursors, for example, cyanide and cyanamide, when exposed to water. If the resulting terrain were not flat, then rainwater while dissolving these reagents would form small streams that combine, mix and drain into pools at the basin (Figure 6C). Given exposure to UV-light, this geological scenario by means of water flow across the landscape could initiate a reaction network which affords the production of ribonucleotides and other relevant molecules.

In 2018, Ritson, Sutherland and co-workers [116] used flow chemistry to help model this geochemical scenario whereby streams of different chemical composition combine and collect. In one variation demonstrated, the flow-chemistry reaction network begins with two separate aqueous streams of (*i*) glycolonitrile and (*ii*) catalytic potassium ferrocyanide, sodium sulfite and inorganic phosphate at pH 6.5. These streams are then mixed and sent through a UV-reactor for irradiation at 254 nm. Like cyanocuprates, mixtures of ferrocyanide and sodium sulfite upon UV-photolysis are also known to generate [117] hydrated electrons that afford the reduction of glycolonitrile to its imine derivative, which can further hydrolyse to glycolaldehyde **4**. At this point in the reaction mixture, the excess sulfite favours the reversible formation of bisulfite adducts. The resulting single combined stream is made to concentrate by dry-down and then passed into a vessel containing solid calcium cyanamide (CaNCN). The calcium precipitates as CaSO_3_, freeing up glycolaldehyde, allowing it to react with cyanamide, which itself is produced upon addition of water, yielding 2-aminooxazole **27**. Very recently Sutherland and co-workers demonstrated [118] an alternative synthesis of cyanamide by oxidative conversion of thiourea using ferricyanide. UV-irradiation of a mixture of potassium ferrocyanide, potassium cyanide and thiourea yielded both cyanamide as well as Kiliani-Fischer reductive homologation products. In addition, 2-aminoimidazole, a structurally similar compound to 2-aminooxazole **27** was also observed.

An alternative approach towards ribonucleotide synthesis involves reaction networks driven by ionising radiation instead of UV light [119]. Exposure of water to ionising radiation, like alpha, beta or gamma rays, is well known to result in hydroxyl radicals (•OH), hydrated electrons and hydrogen atoms (H•) [120]. (It is also worth mentioning that the formation of microdroplets has been recently reported [121] to result in •OH and $e_{aq}^{-}$ production). Radiation chemistry has a long history of being used to model early Earth prebiotic chemistry, starting with Calvin’s 1951 report [122] that radiolysis of aqueous solutions of CO_2_ results in its reduction. Natural nuclear reactors [123] have been proposed as potential geochemical sites that can sustain highly radioactive environments for 10^5^–10^6^ year timescales [124]. The surface of radioactive mineral deposits could also provide relatively high doses of radiation. Draganić and Draganić were perhaps the most prolific in this context [125,126,127,128,129,130] exploring the radiolysis of aqueous solutions of nitriles—HCN in particular—showing that a variety of potentially important molecules like amino acids are produced.

**Figure 7:**
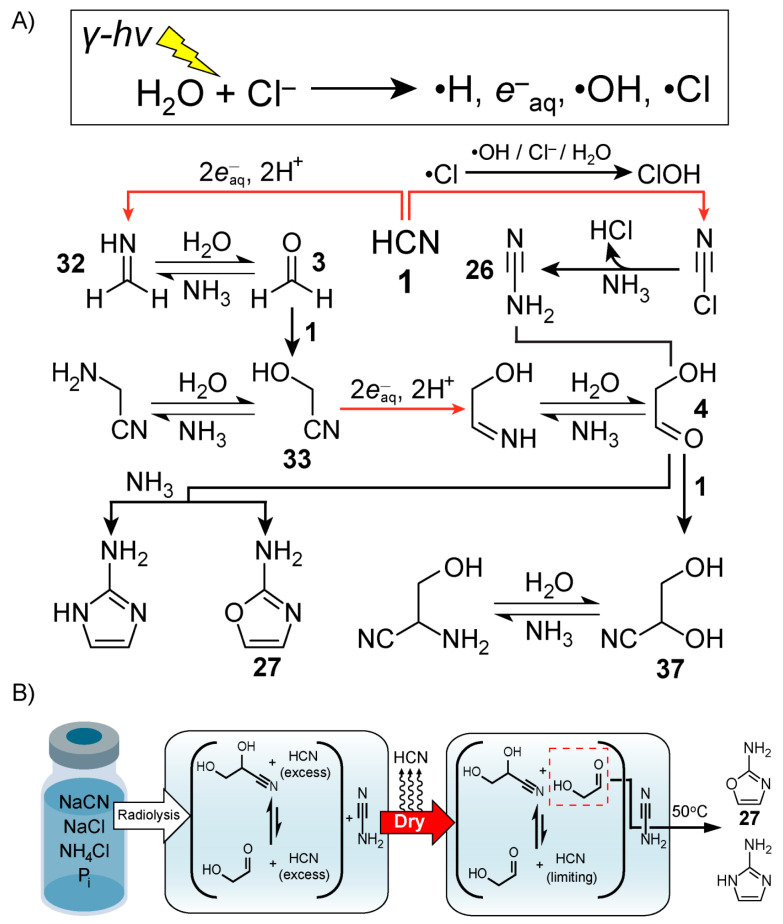
Production of ribonucleotide precursors by γ-radiolysis of briny HCN solutions demonstrated by Fahrenbach and co-workers [51]. (**A**) Radiolysis of briny water produces the reducing and oxidising species required for the synthesis of glycolaldehyde **4** and cyanamide **26** which come together in the first step of the Powner-Sutherland pathway to produce 2-aminooxazole **27**. (**B**) Scheme for the experimental procedure that starts with gamma radiolysis followed by dry-down and heating. Schemes were redrawn based on Figure 2 and Figure 3 from reference [51].

In 2020, Fahrenbach and co-workers [51] demonstrated a continuous reaction network capable (Figure 7A) of producing ribonucleotide precursors relevant to the Powner-Sutherland pathway, particularly 2-aminooxazole **27**, starting from HCN **1** as the only carbon source. The team exploited the redox power afforded by radiation chemistry with a dry-down scenario, in order to generate the needed precursors, i.e., glycolaldehyde **4** and cyanamide. This radiolytic reaction network also produced 2-aminoimidazole, a product which is favoured by increasing concentrations of NH_4_Cl when reacting glycolaldehyde with cyanamide [131]. As shown by Szostak and co-workers [132], this molecule is an effective leaving group in the context of nonenzymatic template-directed RNA synthesis – a potentially prebiotic process for replication of genetic material. Vials of briny solutions containing concentrated NaCl with lesser amounts of NH_4_Cl and P_i_, together with dilute HCN **1**, were exposed to gamma radiation. In the proposed mechanism, gamma radiolysis generates hydrated electrons which initiate a Kiliani-Fischer-type [26] synthesis that affords the production of the cyanohydrins, glycolonitrile and glyceronitrile **37**, from the reversible addition of HCN **1** to formaldehyde **3** and glycolaldehyde, respectively. At the same time, radiolysis of concentrated NaCl solutions produces chlorine radicals (Cl•), which are hypothesised to be involved in the generation of cyanamide [133]. One possible mechanism involves the Cl•-mediated production of HOCl, which reacts with -CN to afford ClCN, an intermediate detected by gas chromatography-mass spectrometry. The NH_3_ in solution reacts with ClCN to yield cyanamide. Control studies revealed that free glycolaldehyde **4** rather than its cyanohydrin, glyceronitrile, is needed to produce 2-aminooxazole efficiently. Following radiolysis, the exposed solution was subjected to a dry-down step, which removed the excess volatile HCN **1**, freeing up a fraction of glycolaldehyde **4** from glyceronitrile by Le Chatelier’s principle (Figure 7B). The dry-down step also serves to increase the concentration of the solutes, which promotes the synthesis of 2-aminooxazole **27** as well as 2-aminoimidazole [131]. No purification/separation or additional feedstocks were employed, and these precursors for RNA synthesis were generated even amongst a diverse mixture of other radiolytically derived products such as formamide, aminoacetonitrile, and glycolic acid. These three compounds are known for their roles in prebiotic nucleotide and peptide production hinting at the possibility of potential chemical co-evolution from a single continuous reaction network.

## 7. Nonenzymatic Analogues and Models of Metabolic Cycles

Genetics-first approaches assume that the initiation of Darwinian evolution by the emergence of a self-replicating molecule like RNA was the key event in life’s history, eventually giving rise to modern metabolism. Therefore, from this perspective, understanding the genesis of the central hubs in modern metabolism ultimately hinges on first elucidating the origin of the initial genetic replicator. Because of this assumption, discontinuous synthesis models for the prebiotic production of nucleic acids, that seem to rely on highly specific scenarios and perhaps chance events, may not be problematic [53]. As long as the abiotic environment can amply provide these compounds while affording nonenzymatic genetic replication, then one of the key origins problems will be solved. Arguably, the emergence of metabolic cycles was then a later product of selection pressures, and not necessarily linked directly to an exclusively abiotic geochemical origin.

Despite the overwhelming evidence of involvement of ribonucleotides in extant biology and the possible explanation of this fact by the prior existence of an RNA world, the hypothesis that RNA originally arose from ancient abiotic geochemistry has received criticism [134]. For example, the presence of RNA in biology today could have been a product of later evolution and does not necessarily reflect a direct role during life’s emergence [135]. In addition, tightly coupled and dynamic reaction networks, like those associated with biological metabolism, appear to be lacking in discontinuous models for prebiotic RNA synthesis, a situation viewed by some as problematic [53]. Metabolism-first approaches to the origins-of-life problem offer an alternative, if not complementary, avenue of investigation. Rather than being a later product of Darwinian evolution, these approaches assume that the universal core of metabolism is closely linked to the abiotic geochemistry from which primitive versions of these networks originally arose. Once this core protometabolic network was established, genetic replicators like RNA emerged later. Hence, metabolism-first scenarios place a greater emphasis [53] on finding nonenzymatic analogues to modern biological metabolic pathways, and tend to prefer continuous reaction networks which more closely resemble the type of chemistry that would have been compatible within cell-like compartments.

The tricarboxylic acid (TCA) cycle has been an attractive target for prebiotic chemistry in this context. Nearly all anabolic pathways in the chart of intermediary metabolism stem from intermediates associated with the TCA cycle [1,136]. Genomic and metabolomic studies indicate the possibility that an ancestor of the TCA cycle was present [1,137,138] during the origin of modern metabolism, while the reverse tricarboxylic acid (r-TCA) cycle affords an anabolic pathway for the fixation of CO_2_—a likely abundant feedstock molecule on early Earth. From a chemistry perspective, the r-TCA cycle is autocatalytic, as long as none of the intermediates are diverted out of the pathway [139]. From a biology perspective, it is a metabolic pathway for CO_2_ reduction that likely evolved before the pentose phosphate pathway [137], providing the five universal metabolites namely, acetate, pyruvate, oxaloacetate, succinate and α-ketoglutarate [136].

**Figure 8:**
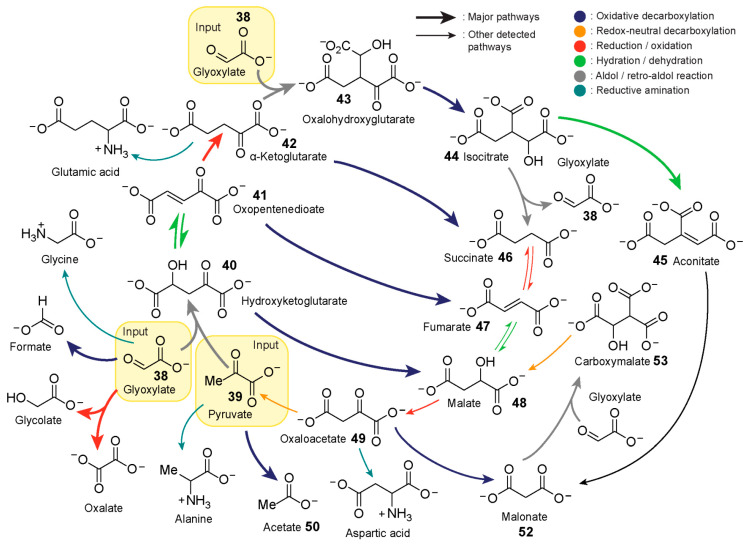
Iron-promoted reaction network capable of synthesis and breakdown demonstrated by Moran and co-workers [23]. The highly interconnected reaction network starting from glyoxylate **38** and pyruvate **39** produced 9 of 11 intermediates of the TCA cycle and 8 of 9 intermediates of the glyoxylate cycle. The addition of hydroxylamine and Fe^0^ to the reaction network affords reductive amination of glyoxylate, pyruvate, α-ketoglutarate, and oxaloacetate yielding glycine, alanine, glutamic acid, and aspartic acid, respectively. The scheme was redrawn based on Figure 1A of reference [23].

In 2019, Moran and co-workers [23] explored the capacity of iron to promote a (r-)TCA cycle analogue starting from glyoxylate **38** and pyruvate **39** (Figure 8). Pyruvate and glyoxylate have been shown to be accessible [140,141] by CO_2_ fixation through abiotic processes. Additionally, theoretical analysis of all known metabolic reactions points to a hypothetical metabolic network where the two ketoacids serve as important hubs [142]. Iron, on the other hand, is one the most abundant metals in Earth’s crust, and large amounts of iron(II) prior to the rise of oxygen is a likely possibility. The authors heated an aqueous mixture of glyoxylate **38**, pyruvate **39** and Fe^2+^ at 70 °C under inert conditions to simulate an Fe^2+^-rich hydrothermal geochemical scenario. Samples from the mixture at different timepoints were taken for analysis by gas chromatography-mass spectrometry and NMR spectroscopy. The data revealed the generation of a highly interconnected chemical pathway capable of both anabolic and catabolic activities by employing five main reaction mechanisms: oxidative and redox-neutral decarboxylation, reduction/oxidation, dehydration/hydration, and aldol/retro-aldol reactions.

The aldol addition between pyruvate and glyoxylate yields hydroxyketoglutarate **40**. This intermediate exists in equilibrium with its dehydrated version oxopentenedioate **41** which can be subsequently reduced presumably by Fe^2+^ to yield α-ketoglutarate **42**. α-Ketoglutarate undergoes another aldol addition with glyoxylate to yield oxalohydroxyglutarate **43**, followed by oxidative decarboxylation, forming isocitrate **44**. Isocitrate can either slowly undergo dehydration to yield aconitate **45** or Fe^2+^-catalysed retroaldol fragmentation, releasing glyoxylate and succinate **46**. Heating succinate under standard conditions leads to trace amounts of fumarate **47**, which itself is in equilibrium with its hydrated version, malate **48**. Malate presumably oxidises to oxaloacetate **49** followed by oxidative decomposition to pyruvate and acetate **50**. The introduction of hydroxylamine and metallic iron (Fe^0^) to the mixture after one hour affords reductive amination of glyoxylate, pyruvate, α-ketoglutarate, and oxaloacetate to yield glycine, alanine, glutamic acid, and aspartic acid, respectively. The resulting reaction network was shown to display significant resemblance to the TCA and glyoxylate cycles, containing 9 of 11 and 8 of 9 intermediates, respectively. The authors speculate that the incorporation of phosphorus and sulfur into the network could provide high-energy molecules capable of polymer synthesis, some of which could possess catalytic properties.

Another important feature for protometabolic reaction networks is their ability for sustained cycle turnover [22]. Rather than trying to replicate the (r-)TCA cycle nonenzymatically from a top-down approach, another strategy involves the bottom-up development of reaction networks based on simple carboxylates that exploit a similar set of reaction types. In 2018, Springsteen, Krishnamurthy and co-workers demonstrated [22] two linked reaction cycles that utilise glyoxylate as the carbon source and H_2_O_2_ as the oxidant. The bicyclic reaction network, linked by their common intermediate oxaloacetate **49**, can be initiated via the aldol addition of glyoxylate **38** with oxaloacetate, malonate **52** or pyruvate **39**.

**Figure 9:**
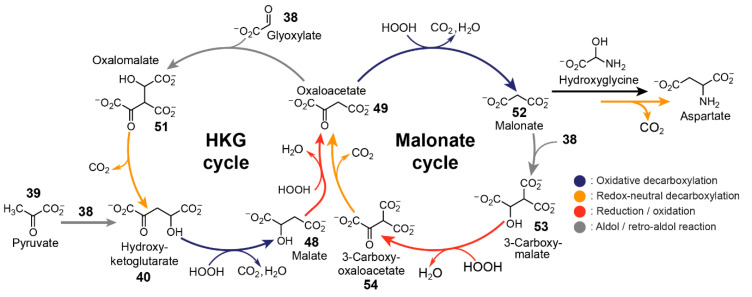
Linked cycles analgous to the TCA which rely on alternating additions of glyoxylate and H_2_O_2_ demonstrated by Springsteen, Krishnamurthy and co-workers [22]. The reaction network can be initiated by the aldol addition of glyoxylate **38** with either pyruvate **39**, oxaloacetate **49** or malonate **52**. Aspartic acid can be generated via the reaction of malonate and hydroxyglycine, formed from glyoxylate and ammonia, followed by decarboxylation in the presence of Mg^2+^. Scheme redrawn based on Figure 2 from reference [22].

The two cycles utilise four types of reaction mechanisms, namely spontaneous decarboxylation of β-ketoacids, oxidative decarboxylation of α-ketoacids, aldol addition with glyoxylate, and alcohol oxidation (Figure 9). The first cycle, referred to as the hydroxyketoglutarate (HKG) cycle, is initiated by the aldol addition of either oxaloacetate or pyruvate with glyoxylate to form oxalomalate **51** or hydroxyketoglutarate **40**, respectively. Hydroxyketoglutarate can also be formed from the spontaneous decarboxylation of oxalomalate. Subsequently, hydroxyketoglutarate **40** undergoes oxidative decarboxylation via reaction with H_2_O_2_ to form malate **48**, which then oxidises back to oxaloacetate. Oxaloacetate can also partake in the second, so-called malonate cycle. The oxidative decarboxylation of oxaloacetate yields malonate **52**, which undergoes aldol addition with glyoxylate **38** to form 3-carboxymalate **53**. 3-Carboxymalate undergoes oxidation to form 3-carboxy-oxaloacetate **54**, which can undergo spontaneous decarboxylation to form oxaloacetate, arriving back at the beginning of the cycle.

While all the steps within a cycle can occur in one pot, glyoxylate and H_2_O_2_ should not be added all at once as glyoxylate can also be oxidised by H_2_O_2_ to form formate. The reaction network was demonstrated to be tolerant to a range of buffers and pH values with most steps proceeding at 23 °C while slower steps were incubated at 50 °C. The authors noted that the conditions employed in this study were relatively mild compared to other nonenzymatic studies of canonical (r-)TCA intermediates. Using isotopically labelled substrates, the authors demonstrated the high turnover potential of the malonate cycle, defined as “the average number of turns of the cycle that occur before the cycle is disrupted” [139]. The same demonstration was not possible for the HKG cycle as the addition of H_2_O_2_ for the conversion of malate back to oxaloacetate also diverts it into the malonate cycle. Furthermore, the team noted that the demonstrated system has the capability to fuel abiotic pathways. For example, the amino acid aspartate was formed when NH_3_ was incorporated in the malonate cycle. According to the mechanism, hydroxyglycine, generated from glyoxylate and NH_3_, reacts with malonate to yield β-carboxyaspartate, which undergoes spontaneous decarboxylation in the presence of Mg^2+^ to yield aspartate.

**Figure 10:**
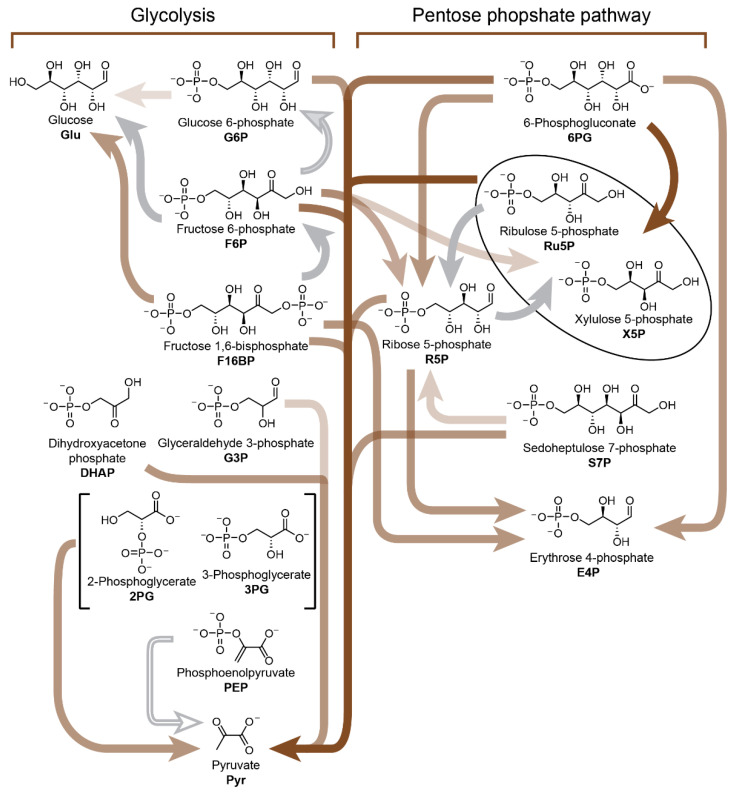
Iron dependency of nonenzymatic reactions in a chemical network reminiscent of glycolysis and the pentose phosphate pathway. Reactions accelerated by iron are coloured with brown arrows while those slowed down are shown with grey arrows. Iron-independent reactions are shown with white arrows. Scheme redrawn based on Figure 4B from reference [146].

While most metabolism-first approaches focus on (r-)TCA cycle analogues, the pentose phosphate pathway and glycolysis are nevertheless metabolic processes common to nearly all organisms. The origins of these metabolic pathways may have been tightly linked to Earth’s ancient geochemistry, in particular the abundance of aqueous Fe(II) evidenced by Archean sediments [143,144], an metal important to a variety of enzymes. In 2016, Keller, Ralser and co-workers reported [145,146] a nonenzymatic reaction network which exploits the catalytic properties of Fe(II) and interconverts sugar phosphate intermediates between pathways that resemble either glycolysis or the pentose phosphate pathway depending on the pH (Figure 10). Using NMR spectroscopy and mass spectrometry carried out on over 4000 samples, the team studied the effect of pH on the Fe(II)-catalysed (but nonenzymatic) decomposition and isomerisation of the various intermediates (e.g., glucose 6-phosphate, fructose 1,6-bisphosphate, ribose 5-phosphate, glyceraldehyde 3-phosphate, etc.) associated with biochemical glycolysis as well as the pentose phosphate pathway. Using this data, a network involving 26 reactions was pieced together. Chemical transformations associated with the pentose phosphate pathway could be trigged by alkaline pH, while those relevant to glycolysis were favoured by slightly acidic or neutral pH. Ferrous iron increases the reaction rates for a majority of the transformations in either nonenzymatic reaction (sub)network. The authors conclude that these nonenzymatic pathways could have been taken over by enzymes, eventually giving rise to the extant glycolysis and pentose phosphate pathways found in cells today.

**Figure 11:**
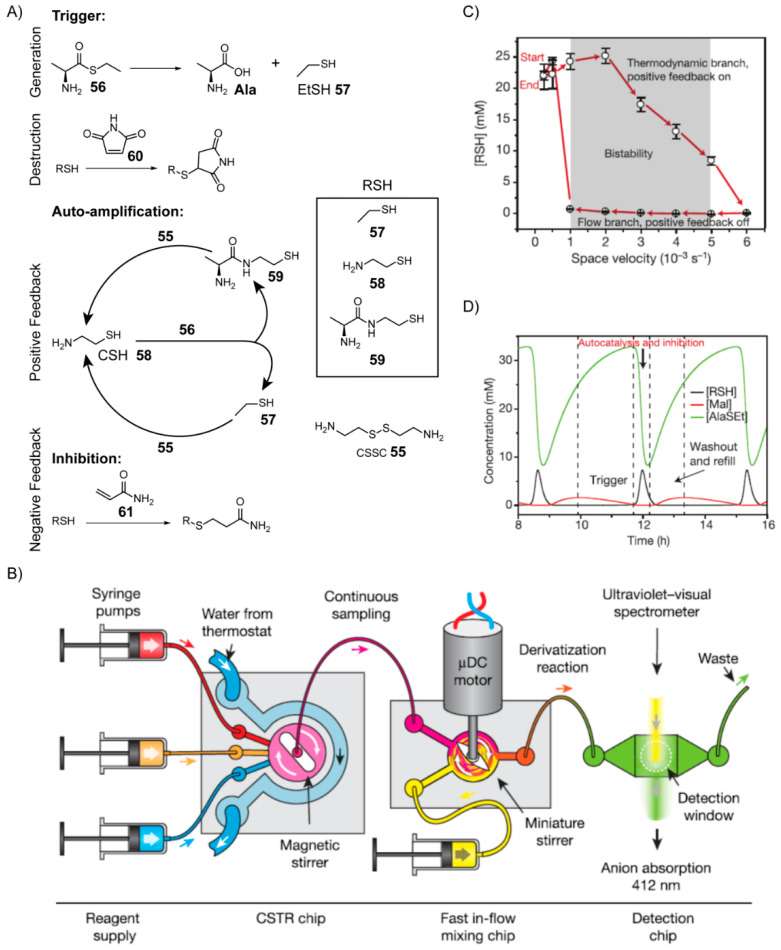
Autocatalytic chemical network consisting of biologically relevant reactions capable of bistability and oscillating behaviours demonstrated by Whitesides and co-workers [147]. (**A**) Scheme of the organic reactions that comprise the autocatalytic chemical system based on reference [147]. (**B**) The continuously stirred tank reactor (CSTR) used to study the emergent properties of the chemical system. (**C**) Hysteresis curve based on experimental steady-state RSH (free thiol) concentrations as a function of space velocities (normalised flow rates). (**D**) A kinetic model simulating oscillating concentrations of RSH, maleimide, and AlaSEt based on experimental observations. Images in (**B**–**D**) were reprinted with permission from reference [147].

In order to study the principles that give rise to emergent behaviours in complex chemical systems, Whitesides and co-workers [147] developed a nonenzymatic autocatalytic reaction network from rational design using chemical transformations relevant to biology and potentially prebiotic chemistry (Figure 11). Here, the goal was not to find a nonenzymatic analogue of an extant metabolic cycle. Rather, the aim was to develop an autocatalytic reaction network, which by implementing out-of-equilibrium protocols could display bistable and oscillatory behaviour in order to better understand these features and elucidate their possible relevance to life’s origins.

To accomplish this feat, the team first engineered an autocatalytic reaction network, which exploited the chemistry of thiols and thioesters. The starting components include cystamine (CSSC) **55** and l-alanine ethyl thioester (AlaSEt) **56** (Figure 11A). The reaction is initiated (triggered) by hydrolysis of AlaSEt to alanine and ethanethiol (EtSH) **57**, the latter of which then reacts with the disulfide CSSC **55** in a thiolate-disulfide interchange to liberate a molecule of cysteamine (CSH) **58** in the process. In comparison to hydrolysis, the reaction of AlaSEt with CSH **58** occurs very rapidly yielding both EtSH and l-alanine mercaptoethyl amide **59**. Both EtSH and l-alanine mercaptoethyl amide **59** react with CSSC **55** via thiolate-disulfide interchange to yield a net total of two equivalents of CSH **58**. Because one CSH molecule reacts to form two copies of itself, the reaction network is autocatalytic after initiation. The team then demonstrated that by including maleimide **60**, which rapidly reacts with thiolates, the onset of the exponential growth phase could be delayed until all the maleimide is consumed. In other words, simply by varying the concentration of maleimide initially included, the timing of the trigger could be controlled.

Next, in order to keep the reaction network out of equilibrium, the team used a continuously stirred tank reactor (CSTR). Such a reactor uses syringe pumps to continuously inject fresh reactants into a reaction vessel, which then allows them to mix and react for some time before exiting the vessel at controlled rates (Figure 11B). Autocatalytic reactions can become bistable when carried out in the context of a CSTR, a feature manifested as hysteresis. In order to detect this hysteresis, AlaSEt **56**, CSSC **55** and maleimide **60** were injected into the CSTR first starting at low flow rates, followed by transitioning to higher flow rates, and then back down to lower flow rates. The concentration of free thiols (RSH) present was monitored just downstream of the reaction vessel after exiting using a spectrophotometer. Low flow rates (measured in terms of space velocity, which is defined as the flow rate normalised by reaction volume, Figure 11C) are required to trigger the autocatalytic amplification and generate a high concentration of free thiols. At higher flow rates, less reaction time is made available, and the concentration of free thiols steadily decreases. Eventually, past a threshold flow rate, the system transitions out of the auto-amplification phase, at which point the concentration of free thiols is negligible. This high flow rate is then ramped back down to the original low flow rate. Since the auto-amplification process has already been deactivated at this stage, the concentrations of free thiols remain below the necessary threshold, until the flow rate is slow enough again to afford sufficient reaction time and initiate the trigger (by reacting with all the maleimide). This observed hysteresis behaviour is akin to a type of “memory” that can emerge spontaneously from autocatalytic reaction networks. Usually memory is associated with genetic polymers like RNA, rather than dynamic processes.

Bistable systems can also yield periodic oscillatory behaviour given the presence of an inhibition mechanism that can slowly remove the species undergoing auto-amplification. The authors found that when acrylamide **61** was included in excess, a compound which reacts with thiols more slowly than any other member of the reaction network, periodic oscillatory behaviour in the concentration of free thiols is observed (Figure 11D), at least in the context of a CSTR within a certain range of flow rates. This oscillatory behaviour can be explained by three distinct stages. In the first stage, there is a delay setup by maleimide **60** which must first be depleted. The next stage involves autocatalytic amplification of free thiols, which competes with the inhibition reaction caused by acrylamide **61** until all of the AlaSEt is depleted. In the third stage, the depleted AlaSEt and other reagents are replenished by continuous flow into the system, regenerating the conditions needed to begin another round of oscillation. Hence, this autocatalytic reaction network without any enzymes exhibits emergent behaviours similar to biological systems and suggests the possibility that such behaviour could have resulted abiotically on the early Earth.

## 8. Conclusions

Recent research efforts have demonstrated progress in prebiotic reaction networks across multiple fronts, including analytical, theoretical and experimental strategies. Metabolomics techniques like Van Krevelen diagrams and Kendrick maps can also allow for broad characterisation of prebiotic reaction mixtures. In terms of theoretical predictions, graph grammar computational protocols are a powerful analytical tool for the discovery of new autocatalytic pathways hidden in experimental data. Even without computer-assistance, autocatalytic cycles can be discovered and even rationally designed. The formose reaction is a classical example of autocatalysis having led to new pathways for ribose production exploiting borate minerals, which resulted in proposed discontinuous pathways for ribonucleotide synthesis and highlights the need for more investigations of minerals in the context of prebiotic chemistry. Alternative routes that avoid direct ribose production altogether, building up both the sugar and nucleobase components concurrently, have been shown, and at least some of these steps can occur in a continuous manner. Meanwhile, inspired by metabolism-first ideas, the search for nonenzymatic analogues of the biological (r-)TCA cycle and other pathways has seen much progress. The roots of glycolysis and the pentose phosphate pathway may trace back to abiotic iron chemistry. Examples of reaction networks utilising TCA cycle substrates promoted by transition metals as well as oxidising agents have been realised, however, a complete autocatalytic cycle has yet to be demonstrated. Nevertheless, it is indeed possible to engineer autocatalytic reaction networks using small molecules and mechanisms relevant to biology and possibly prebiotic chemistry, leading to bistable and oscillatory phenomena reminiscent of life-like behaviour.

Reaction networks have spanned both continuous and discontinuous models, under the presumptions of both genetics-first and metabolism-first approaches, perhaps revealing that an integrated approach is not far off. But there is still likely a long way to traverse before we are able to replicate something as complex as the cell in the lab. What are the most promising future directions? We believe the future of prebiotic chemistry lies in the continued development of new experimental, theoretical, and analytical protocols that can aid in better understanding of complex reaction networks.

Prebiotic chemistry would benefit greatly from the establishment of centralised databases that parallel biology-focused ports such as Reactome, EMBL-EBI, ENSEMBL, etc. This belief is partly inspired by the approach utilised by molecular biologists as a means to share data acquired from limited samples and streamline data analysis. This proposition, which aligns with outcome 2.1.3 of “*A Strategy for Origins of Life Research*” by Scharf et al. [148], involves the construction of a centralised peer-reviewed database that would allow for collaborative communication between theorists and experimentalists across multiple disciplines. An example of this concept already has been realised in the recently published computational study by Grzybowski and co-workers [38]. The team constructed a network in silico using a forward-synthesis algorithm using a set of rules based on prebiotic reactions reported in the literature. Information on structural-motif reaction conflicts and reaction conditions, such as solvents and temperatures, were also exploited by the network algorithms. Randomly chosen pathways generated by this “computer-assisted organic synthesis” protocol were plotted on a graph that visualised the changes in pH necessary at each step. Such representations provide an example of how to evaluate the number of discrete intervention steps required for a particular subset of reactions in a network, and hence, where a reaction network lies along the continuous-discontinuous spectrum. In addition, three forms of chemical emergence, supported by experimental data, were demonstrated, a notable example of which is a novel autocatalytic cycle for iminodiacetic acid. This database (https://life.allchemy.net) has been made freely available to the community.

Further development of these types of databases that will facilitate the combination of theory and experiment possess great potential to expand our knowledge in prebiotic reaction networks. Such databases will allow for network-level analyses of increasingly greater sophistication to take place, like the ones that have already been applied to biological reaction networks, which reveal their underlying non-random structures and attributes. Recently, an analysis of compiled data mostly from radiolytically generated reactions revealed [149] network-level topological attributes that are associated with the traits of evolvability and self-organisation, properties of which also are crucial for cell biology and ecosystem ecology. The question remains an open one, whether living systems can arise only from abiotic chemistries that already possess the network organisational features observed in current biology, or if these attributes arose later as a consequence of selection, survivability or enzyme takeover [149]. These sorts of topological analytical methods applied to realised complex reaction networks could lead to the innovation of much needed new perspectives for deconstructing how, when and where abiogenesis happened on Earth and perhaps other worlds [150]. These innovations would allow the field to progress beyond the “I know it when I see” style of categorisation to more quantitative and precise descriptions of prebiotic reaction networks in water.
